# ‘An uneasy compromise’: strategies and dilemmas in realizing a permissive abortion law in Ethiopia

**DOI:** 10.1186/s12939-019-1017-z

**Published:** 2019-09-27

**Authors:** Getnet Tadele, Haldis Haukanes, Astrid Blystad, Karen Marie Moland

**Affiliations:** 1 Department of Sociology, Addis Ababa University, Addis Ababa, Ethiopia; 2 Department of Health Promotion and Development, University of Bergen, Bergen, Norway; 3 Global Health Anthropology Research Group, Centre for International Health, Department of Global Health and Primary Care, University of Bergen, Bergen, Norway; 4 Centre for Intervention Science in Maternal and Child Health (CISMAC), University of Bergen, Bergen, Norway

**Keywords:** Abortion, Policy, Access, Silence, Ethiopia

## Abstract

**Introduction:**

At the turn of the century, when the Millennium Development Goals placed maternal mortality reduction high on the global agenda, Ethiopia relaxed its restrictive abortion law to expand grounds on which a woman could legally obtain an abortion. This radical policy shift took place within a context of predominant anti-abortion public opinion shaped by strong religious convictions. Drawing upon Walt and Gilson’s policy analysis framework, this paper explores the tension between public policy and religious dogma for the strategies chosen by the Ethiopian Ministry of Health and its partners implementing the new policy, and for access to safe abortion services.

**Methods:**

The study employed a qualitative research methodology. It targeted organizations that are key stakeholders in the field of reproductive health. These included policy makers and policy implementers like ministries, UN agencies and international and national NGOs as well as religious organizations as key opinion leaders. The data collection took place in Addis Ababa between 2016 and 2018. A total of 26 interviews were conducted, transcribed, and analyzed using the principles of qualitative content analysis.

**Results:**

Our analysis showed that the implementing organizations adopted a strategy of silence not to provoke anti-abortion sentiments and politicization of the abortion issue which was seen as a threat to the revised law and policy. This strategy has facilitated a rollout of services and has improved access to safe abortion care. Nevertheless informants were concerned that the silence strategy has prevented dissemination of knowledge about the revised law to the general public, to health workers and to the police. In turn this has caused confusion about eligibility to legal and safe abortion procedures.

**Conclusions:**

While silence as a strategy works to protect the law enhancing the health and survival of young women, it may at the same time prevent the law from being fully effective. As a long term strategy, silence fails to expand awareness and access to safe abortion services, and may not sufficiently serve to fulfill the potential of the law to prevent abortion related maternal deaths.

## Introduction

At the turn of the century, when the Millennium Development Goals placed maternal mortality reduction high on the global agenda, Ethiopia relaxed its restrictive abortion law to expand the grounds on which a woman could legally obtain an abortion [1]. The revision of the abortion law in Ethiopia was negotiated in a strongly anti-abortion cultural and religious environment [2] and, although Ethiopia is formally a secular state with constitutional freedom of worship, religion is very much present in the public sphere and in community life [3]. As this paper will show, the historically dominant Ethiopian Orthodox Tewahido Church (EOTC) has a strong hold on public opinion and, more than a decade after the enactment of the law, 83% of EOTC members perceived abortion as morally wrong [4]. This paper takes a closer look at the law and how it has fared during the period it has been operational, paying particular attention to the tension arising at the intersection between a permissive law and a religiously conservative society.

Induced abortion is legally restricted in most of Africa. The only countries where abortion is broadly legal are South Africa, Tunisia and Cape Verde [5]. The grounds on which induced abortion is allowed vary from severely restrictive, where abortion is only allowed to save the life of the mother (e.g. Tanzania), to abortion on social and economic grounds (e.g. Zambia). In Ethiopia, induced abortion was, until 2004, illegal in the strict sense and was only permitted ‘to save the pregnant woman from grave and permanent danger to life or health’ ([6] Art. 534). With a maternal mortality ratio of 743 per 100 000 live births, Ethiopia had at the time one of the highest rates of maternal death in the world [7]. It was estimated that abortion-related complications accounted for 32% of all maternal deaths in 2005 [7]. Furthermore, the burden on the health system and the cost of care to treat abortion complications was very high [7]. It was in this maternal health and health systems context that the revised abortion law was fought through in 2004.

Since religion and religious beliefs are central in the lives of Ethiopians - the majority of the population is Christian (62%,) or Muslim (34%) - the initiative to liberalize the abortion law met strong opposition. Although membership of the protestant and evangelical churches is on the increase, EOTC is the biggest religious community constituting 44% of the total population [8]. Despite being separated from the state since 1974, the EOTC continues to hold moral authority, shaping ideas about right and wrong in society. The Patriarch of the church, as well as the local priests, are highly influential in family affairs, not least in matters concerning life and death, and sexuality and reproduction [9]. Thus, in the years preceding the revision of the law, Ethiopia experienced intensive resistance from anti-abortion groups consisting of religious leaders, gynaecologists, lawyers, and others who perceived that the law would violate religious rules and moralities [2]. The Christian Workers’ Union for Health Care, an important actor in the debate, had ties to the global pro-life movement mainly based in the US. Their major arguments were that abortion is strictly forbidden in the Bible and that safe abortion is a myth – all abortions are unsafe and will cause different types of physical and psychological trauma. Their position was supported by the Patriarch of EOTC who disseminated the message in the newspapers, arguing that abortion is inhuman, selfish and against the divine commandment not to kill [2].

Advocates for liberalisation of the law, spearheaded by the Ethiopian Society of Obstetricians and Gynecologists (ESOG), and supported by international and local NGOs, nonetheless advanced revision of the abortion law on the grounds of the magnitude of unsafe abortions in the country and its severe consequences for women’s health and wellbeing [2]. Hence the revision of the law was argued as part of the national effort to bring down maternal mortality and to achieve the aims of the UN Millennium Development Goal 5 (MDG5).

The revised law, enacted in 2004, allows women to terminate pregnancies that result from rape or incest; if the woman has physical or mental disabilities; if continuation of the pregnancy or the birth would endanger the health or life of the woman or fetus; if the fetus has incurable disease or deformity; and if the woman is a minor who is physically or mentally unprepared for childbirth ([1]: Article 552:1).

Even though the law went a long way towards liberalization, abortion is still classified as illegal in the country’s revised Criminal Code [1] and is punishable by imprisonment if violated. Abortion thus remained a criminal act with a few exceptions, but the law also settled that “the mere statement by the woman is adequate to prove that her pregnancy is the result of rape or incest” ([1]: Article 552:2). This was a radical statement that went a long way in shifting the responsibility for the decision to terminate a pregnancy from the police and the health worker, to the woman herself. The Ministry of Health was mandated to produce Technical and Procedural Guidelines for Safe Abortion Services which were issued in 2006 in a tone of gender equality and choice with reference to international treaties on human rights and women’s rights as well as national policies on women and children [7]. The guidelines were meant to ensure that all women obtain standard, consistent, and safe termination of pregnancy services as permitted by the law [7]. Interestingly, in operationalizing the law, the ministry added that ‘the provider will use the stated age on the medical record for age determination to determine whether the person is under 18 or not. No additional proof of age is required’ ([7]: 10). The guidelines further state that, ‘The provider should, in all good faith, follow the knowledge of standard medical indications that necessitate termination of pregnancy to save the life or health of the mother’ ([7]: 10).

A decade after the law was passed, abortion-related maternal deaths were estimated to constitute 19.6% of all maternal deaths in 2013 [10] as compared to 32% before the law was revised [7]. Availability of eligible public and private health facilities that provide safe abortion services was reported to have increased dramatically from 898 in 2008 to 4,033 in 2014, and the proportion of abortion care provided in the public sector was estimated to have increased from 36 to 56% nationally [11]. The proportion of abortions occurring legally in health facilities reportedly increased from 27% in 2008 to 53% in 2014, and the rate of legal abortions more than doubled from 5.8 to 14.7 per 1,000 women [11]. Although these figures indicating an impressive change between 2008 and 2014 may be overrated, great improvements in access to safe abortion services are reflected in reduced abortion related mortality. Still, despite the apparently substantial achievements, the estimations indicate that 47% (294,127) of abortions still occurred outside of health facilities in 2014, and were thus categorized as unsafe. These high rates of unsafe abortion were attributed to lack of knowledge of the law, perceived cost of services, lack of knowledge about service availability, fear of stigma and lack of services in accessible sites. There is also substantial regional variation, and the safe abortion rate continues to be lowest in the least densely populated and peripheral regions. While the vast majority of the Ethiopian population (80%) lives in rural areas, only 39.5% of women accessing abortion care in 2014 were rural residents [11]. This demonstrates a huge urban bias in the roll out of services, partly associated with the concentration of international NGOs providing safe abortion services in urban centres.

The revised abortion law has been called an ‘uneasy compromise’ [12] between the public health concerns of the government working to bring down maternal mortality on the one hand, and religious organizations allied with the protestant Christian Workers’ Union for Health Care in Ethiopia, and pro-life groups with international ties on the other. Using Shore and Wright’s [13, 14] perspective of the ‘social life of policy’ we explore the implications of this ‘uneasy compromise’ for the strategies chosen by actors in the field and for access to safe abortion services. Shore and Wright [14] point out that policies are ‘contested and reshaped’ by the numerous actors involved in the process of implementing and interpreting the law. The abortion law put in place, and the Ministry of Health’s interpretation of the law in the operational guidelines of 2006 and the later version of 2014, is subject to interpretation by actors that are directly or indirectly involved in its implementation and, as we shall argue, this interpretation re-shapes the policy. Following Walt and Gilson [15] and Walt et al. [16], we focus, not primarily on the content of policy, but on the actors involved, the process of implementing the abortion policy in the health system, and not the least on the context of the policy reform. Specifically, we investigate how the revised law and its guidelines, and the implementation process are perceived by key stakeholders in the field of reproductive health and rights, and other actors with vested interests in the matter. Of particular interest are tensions emerging at the interface between a permissive law and a culturally/religiously conservative society, the strategies adopted by actors supporting and opposing the law, and the implications of these for women’s access to safe abortion services.

## Methods

This article reports on one of three country cases in the project entitled ‘Competing discourses impacting girls’ and women’s rights: The case of fertility control and safe abortion in Ethiopia, Tanzania and Zambia’. The Ethiopian case study analyzed here targeted organizations and institutions that are key stakeholders in the field of reproductive health acting as policy makers/advocates for policy change and implementers including ministries, international and national NGOs and UN agencies. Importantly, the study also included religious organizations as key opinion leaders in the field of reproductive health. We used a qualitative interview design to explore experiences and perceptions related to the law and its implementation.

### Data collection

A total of 23 organizations were included in the study. Twenty six interviews (including three repeat interviews) were carried out by the four co-authors (22 interviews) and an MA student from the Department of Sociology, Addis Ababa University (AAU) (four interviews) in three rounds in Addis Ababa in November 2016, March 2017 and March–April 2018. We made a list of eligible ministries, UN agencies, international and local NGOs, professional associations and religious organizations that we wished to interview, and produced an interview guide and an information letter to explain the purpose of the study. A local gender specialist research assistant, who is centrally located in the field of reproductive health in Ethiopia, helped the authors in accessing the organizations and arranging appointments. She contacted the organizations first by phone and later by e-mail with the information letter and interview guide attached. Based on their responses, she developed an interview schedule for the team. Apart from a few that did not respond, the study participants were enthusiastic about sharing their experiences and perceptions of the law and its implementation.

Altogether 23 organizations were included in the sample. The organizations interviewed include: Five ministries (Ministry of Health, Ministry of Education, Ministry of Justice, Ministry of Youth and Sports Affair, Ministry of Women and Children); three United Nations (UN) agencies (United Nations Population Fund (UNFPA), World Health Organization (WHO), UN Women); two professionals associations (Midwives Association and Ethiopian Society of Obstetricians and Gynecologists); one professor from School of Public Health, Addis Ababa University; four International NGOs (Engender Health, Pathfinder, IPAS, Marie Stopes International), four local NGOs (Family Guidance Association of Ethiopia (FGAE), Ethiopian Women’s Lawyers Association (EWLA), Organization for the Development of Women and Children in Ethiopia (ODWACE), Women Health Association of Ethiopia); five religious organizations (Ethiopian Inter-faith Forum for Development Dialogue and Action (EIFDA), Ethiopian Islamic Affairs Council, Ethiopian Evangelical Church, (EEC), Ethiopian Orthodox Tewahdo Church and Ethiopian Orthodox Tewahdo Church Development Commission- EOTCDC). Informants from FGAE, Pathfinder, and IPAS were interviewed twice (in 2016 and 2017) to follow-up emerging issues from the first round of interviews. The organizations had appointed one to three individuals to take part in the interviews. Twenty two of the interviews were conducted by pairs of researchers in English and usually took between 60 and 90 min. Four interviews were conducted in Amharic by a MA student supervised by first author G.T.

### Data analysis and ethics

All interviewees had read the information letter that was sent to the organization beforehand and consented to participate in the study. Among the 22 interviews conducted by the authors of this paper, 20 were audio recorded with the consent of the interviewees while two preferred not to be recorded and the interviewers wrote detailed notes. The recorded interviews were later transcribed verbatim by an experienced assistant. The four interviews conducted by the MA student were audio-recorded, transcribed and translated into English in a summarized form. The transcripts, the English summaries and the notes from the two interviews not transcribed were analyzed by the first authors assisted by the co-authors using the principles of content analysis. This process involved reading and re-reading the transcripts to become familiar with the data, coding the data material, identifying categories and defining themes drawing upon the perspectives of Shore and Wright [14] and the policy analysis frameworks of Walt and Gilson [15].

In order to protect the identity of the interviewees, individual statements have been anonymized, and the organization to which the person belonged is indicated only in categories. Exceptions to this rule are made when the name of the organisation is needed to make sense of the quote. The categories are ministries (MIN), UN agencies (UN), international NGOs (INGO), local NGOs (NGO), professional associations (PA) and religious organizations (RO). The project was approved by the Department of Sociology, Addis Ababa University, by each of the organizations included in the study, and by Norwegian Centre for Research Data (NSD project number 57089/3/00SIRH).

### Findings

Our analysis showed how different actors positioned themselves in the abortion landscape after the revision of the law, how they perceived the development after the law was passed, and how they developed strategies to protect their interest in the field. Silence was identified as a strategy used by actors on several levels, and below we describe how this was enacted and expressed, and what kinds of challenges this strategy implied for access to safe abortion services. Firstly, we look into the various improvements the law has spurred, as narrated by actors centrally situated in the field.

### How the abortion landscape changed after the liberalization of the law

Not surprisingly, the law was praised by many of our informants from the government and non-government sectors for being progressive and opening up different pathways to access safe abortion:

The law is one of the progressive abortion laws in Africa. Although it is not on demand, more or less every woman who requests safe abortion can access the service. As much as possible barriers to services are reduced. (INGO)

Not the least, the positive implications of the law for victims of rape were emphasized: *Yes, that law has significantly changed the way clients are getting safe abortion services,* said an informant from an International NGO. Prior to the revision of the law, the rape victim had to go through a long probation process in court and only if the court supported her case, would she be able to return to the clinic to terminate the pregnancy. In the meantime, the pregnancy advanced, making it more difficult and more risky to get an abortion*.* The clause in the present abortion law about the woman’s statement of rape or incest to qualify for abortion services was seen as critical for provision of timely services: ‘*But now [that] the word of the client is enough, she doesn’t need to go to the court, to the police and so on, that makes it [the process] very rapid*. (INGO) Likewise, stated age as qualification for age-based abortion was considered a vital tool to address unwanted pregnancy among young girls. The law and the guidelines also served to instruct and justify actions taken by health workers and were referred to as *‘our bible’* telling the health workers ‘*on what level and on what criteria to provide the service’*. (NGO)

Access was also said to have improved because of the efforts of the government and non-government actors to enhance infrastructure, through expanding the number of health centres and midwives particularly in rural areas, and by task-shifting abortion care to mid-level professionals.

Previously, it was a board of three physicians who had to approve the service, now one mid-level provider is enough and he/she does not have to be a doctor. The guideline has also eased requirements about who could provide safe abortion. In the past, it was the doctor’s job to carry out abortions (clandestine abortions not taken into consideration), but the guideline stipulates that mid-level providers (nurses, mid-wives, clinical officers) could provide both medical and surgical abortion. (INGO)

One informant from the INGO sector described the developments in the following way:

For a long time, abortion services revolved around the Marie Stopes clinics, the pioneer in providing safe abortion. But now it is provided in almost all health care institutions. I felt the difference between the time when I started my career ten years ago and now. Now, if you randomly go to a health centre, you can find that the service is being provided and, relatively speaking, the service providers are not being stigmatized as they were before. Therefore, abortion is becoming more acceptable. (INGO)

A major impact of the liberalization of the law as experienced by the Ministry of Health and service-providing institutions was that septic abortions were almost done away with. *‘Previously we used to encounter highly complicated cases with severe infections, with injured organs and so on due to unsafe abortion’*. (INGO). This change was demonstrated by the fact that hospitals around the country had closed their ‘septic rooms’ which had previously been used frequently for severe abortion complications. Echoing the above assertion, an official from the Ministry of Health also noted that maternal mortality due to abortion had gone down dramatically.

### Silence to protect the law and avoid confrontation

These improvements in access to safe abortion care and health outcomes could not have been achieved without the revised law, but as one of our informants put it, *‘changing the law is not enough, changing the guideline is not enough’*. (PO) The actors involved in implementing the law and the strategies they developed to extend services without attracting public attention in a predominantly anti-abortion environment seemed to be vital in this process. Although the resistance that arose in the law revision process was no longer loud, many of the proponents of a liberal abortion law and safe abortion services acknowledged the risk of backlash. In order to circumvent upheavals, they kept a low profile and avoided confrontation with groups that were displeased with the change in the law. Many of our informants noted that there is no public strategy on creating demand or advocating for safe abortion services since, as they told us, *‘public opinion is dominantly against abortion’*.

We don’t speak publicly about abortion, we don’t have any media intervention. If you go to the Ministry of Health, they don’t want to talk much about abortion, but do it silently. (INGO)

For the same reason, most of the actors involved in reproductive health in the country also seem to have chosen not to frame abortion as a rights or gender issue, fearing that this would be counter-productive to their cause. Even though the guideline was, as mentioned above, framed in the language of gender and rights, the discourse chosen by government and non-government actors was that of public health and they packaged their messages very carefully:

We don’t directly talk about abortion law, we don’t confront religious groups. Even when they have negative speeches, we don’t want to respond directly. We seek opportunity to speak about the magnitude of unsafe abortion packaging the message. We talk about reproductive health, we talk about maternal mortality and we go in detail about the causes of maternal mortality, then people start to talk about unwanted pregnancy and then they talk about unsafe abortion, these are our approaches at the community level. (INGO)

Hence the entry point to conversations in the community was reproductive health and the terms used by the actors to discuss unwanted pregnancy and abortion with the community were carefully chosen in an attempt to make the messages culturally sensitive and encourage people to talk about sexuality and reproduction.

We don’t promote abortion because the nation is a very conservative society, people don’t talk openly about sexuality. We don’t use the word abortion in Amharic, rather pregnancy termination (*Tsense Maquaret*). The word abortion itself is stigmatizing, the Amharic equivalent is *Wurja*, literally meaning abortion, but we don’t use this term. (INGO)

Because of the sensitive nature of the topic, awareness creation about the guideline was very limited. As one informant commented:

We cannot gather people and tell them this is the new guideline; it is difficult to share the guideline with the media. We focus on practical ways of addressing demand, if we openly talked about it, it may backfire. (INGO)

Our informant from the Ministry of Health also endorsed this argument and said that they don’t talk about the law.

For example, if you are looking at South Africa it is legal but the service is very limited. Here in Ethiopia, the providers are doing it quietly.… keeping silent made the resistance less. They don’t say anything in public…. The commitment of the ministry is to do that silent work. We are very supportive, silently. (MIN)

An informant from a UN organization reiterated the importance of working quietly. She said that they don’t carry out promotion or activism at the community level or through media about where abortion is available and how it is provided.

If we do a promotion activity or activism, the resistance will come especially from the conservative part of the population, religious people will rise. As it is, we are getting the results we want, so I don’t see the need for any more promotion or activism. (UN)

The organizations working to increase access to safe abortion services, based on the provisions of the law, expressed very clearly that the aim of the silent approach they had chosen was to protect the law. As argued by one of these organizations’ representatives:

We work to protect the law. Unless we protect the law, there might be some opposition groups from abroad or from inside the country. We scan the environment; we have regional abortion technical groups in four major regions, five or six persons from different fields. So we scan the media, events and we also scan different speeches and document that. We analyze it and if it has continuity, we see it together with our partners and plan for a strategy on how to respond. (INGO)

At present, there seems to be little opposition to the law. Anti-abortion, or pro-life groups as they commonly call themselves, though registered with a home-page on the internet, show very little activity. This was confirmed by our informants: *‘We have not that much strong opposition like other African countries such as Kenya, Nigeria and Tanzania where funds from abroad create strong opposition’* (INGO) and as summed up by one of our informants: *‘The silence is the secret behind the success’*. (INGO).

### Absence of a confrontational strategy to restrict the law by religious leaders

A major concern for organizations working to extend safe abortion services to eligible girls and women, as defined by the law, was not to provoke anti-abortion sentiments and public reactions, including reactions from religious leaders. An important question was how this strategy was met by religious leaders. Our informants from religious organizations did not speak directly about the process of revising the law, but they did demonstrate their resistance to the law in very specific ways. When asked about his knowledge of the law, one religious leader from EOTC admitted that he did not know the law very well, but he nevertheless rejected it:

I do not have awareness of the abortion law. The government can pass whatever law and it can also do whatsoever possible to enforce the law, but it cannot force the church to change its firm position against abortion. According to the EOTC, abortion is completely prohibited and it should not be allowed. No one can force the church to change this firm stand, because the church has long been autonomous and respected. The church is governed by the resolutions of the synod, not by the law of the government. My knowledge of the law, therefore, does not change anything. (RO)

An Islamic religious leader made a similar statement:

….I think abortion is allowed by the law. No matter what the law says, we do not ask why it is allowed. The government makes laws and as a religious organization we have our own perspectives about it, and our perspectives about abortion are as mentioned thus far [it is a sin and should not be permitted]. … Abortion should not be regarded as a matter of women’s rights, as the life of the mother and that of the child both belong to Allah….. The Ethiopian Islamic Affairs Council has not rejected the law, but gives recognition to only one ground on which abortion can be provided- to save the life of the mother. (RO)

Interestingly, this Islamic leader admitted that his organization has not rejected the law officially. In the same vein, the Ethiopian Evangelical Church (EEC) did not officially reject the law. As one of the leaders stated:

I have awareness of the newly revised abortion law. There is no official stand by the EEC concerning abortion. No official stand means we have not objected to the law, and it may also imply that the church has reservations about it. The law should not be regarded as an opportunity for women to practice their rights to terminating an unwanted pregnancy. The life of the fetus has a divinity connotation. God created humans and the life of human being is honorable. Terminating this life is only the sole right and power of God. Abortion is a breach of the relationships between God and his creatures and the power interplay between man and God. Breaching these relationships by man is a sinful act. Therefore, a woman should not simply jump into making decision to terminate pregnancy, she should think time and again before making this decision. (RO)

Even though all the religious organizations included in our sample had a very clear stand against abortion, they had not voiced their opposition to the law officially after its enactment, and did not seem to foster a political debate to restrict the law. Their strong stand against abortion as a violation of God/Allah’s commandments was communicated through priests and sheiks down to the community level but did not seem to result in a confrontational strategy vis a vis the federal law.

### The limitations of silence as a strategy to extend access to safe abortion services

The limited public debate and limited voiced opposition against the law indicated above, were seen as closely linked to the silent approach adopted by the actors working to secure access to safe abortion services within the law. While this strategy seems to have worked effectively in terms of preventing confrontation, its limitations are clear. The silence has also hindered the law and its guidelines from becoming known. Women, especially in rural areas or in regional towns, therefore lack knowledge about the law and have little access to information that safe abortion services are available through the health system. In a situation where abortion is surrounded by societal silence, religious actors can, within their own domain, pursue a discourse on abortion as a sin and as a moral transgression, meeting very little opposition. According to our informants, women tend to believe that ‘*abortion is illegal on all grounds’* and do not know where to seek help if they experience an unwanted pregnancy. Hence, as emphasized by some informants, safe abortion services offered through public health services at primary care level still tend to be under-used because of lack of knowledge, especially in rural areas.

One of our informants from an INGO told us:

There are recently conducted studies on abortion stigma and we were trying to investigate community-level barriers to women’s access to safe abortion services. We asked the women if they knew about the abortion law of the country and only 48% knew about the law. However, when we go deep down and ask them some of the broad indications of the abortion law, only very few of them knew about the provision. Therefore, information about the abortion law is not widely disseminated to the women though it has progressed over the past ten years. (INGO)

Lack of information about the law was voiced as a problem not only for potential service users but also for health care professionals including midwives, physicians and nurses. *Among the health professionals themselves, there is misunderstanding about the law*, a representative from a professional organization told us. *That is why the nurse said to a girl [a victim of rape] who appeared for safe abortion and related service, “I will take you to the police”[to report the case].* (PA)

Our informant from a local NGO also noted:

We still are witnessing the fact that some service providers do not know the conditions and the entitlements of young people for the services. The challenge now is that many young people are not getting enough information on policy and technical procedural guidelines. We should not be very much deceived by what we see in Addis. A good number of young people are lacking information about this in the country. (NGO)

The same lack of knowledge about the law was seen among other civil servants. According to informants in the NGO sector, even the police and others in the criminal justice system lack knowledge about the law and the procedural guidelines. We experienced this knowledge gap ourselves, when interviewing a high-level official from the Ministry of Justice who demonstrated lack of awareness about the amendment in the law and said that a woman’s word that she was raped is not sufficient to qualify for abortion. He said:

If a woman is raped, she should report to the police station to get safe abortion service. If she goes directly to the health facility for abortion purpose without reporting to the police, she can’t get the service…. She should report to the Ministry of Justice or police to make an investigation. Then, the abortion process will start after the prosecutors prove that she is raped. If health care facilities provide an abortion based on the word of a woman, it is not the proper way. (MIN)

The limited information of the law seemed to reinforce an anti-abortion sentiment among health workers and the public alike, and was seen to sustain the stigma associated with abortion and abortion providers. The silent strategy was not considered helpful in addressing this problem since *‘it is difficult to fight stigma without talking about it’.* Health workers trained in safe abortion care and deployed in their rural home area commonly experienced stigma and found it difficult to provide the services. As an informant from the INGO sector explained to us:

People can easily identify them as abortion care providers and they say a lot of things about them like ‘*you are the baby killer’*. Some even go to their husband and wife and they might hear it. They sometimes come on [confront] them by their religion by claiming it is against religion. (INGO)

The role of health workers as gate keepers to safe abortion services was an issue that was raised by several actors. According to our informants from the service-providing organizations, some providers and facilities resist giving safe abortion services since it goes against their religion and their professional ethos of saving lives. Hence they use their discretionary power to deny services.

They make their own judgment and when they feel that she [the woman seeking abortion services] might not be telling them the truth, they may say ‘you don’t qualify for the service and we will not give you’ [the service]. (INGO)

Rather than denying the abortion seeker services altogether, some health workers would, according to the same informant, avoid providing abortion services by suggesting an alternative procedure: *Some advise the client to go and buy medication abortion and return [for post abortion care] if they see bleeding.* (INGO) In this way the health worker would assist the woman and provide post abortion care to ensure health and survival without playing an active part in inducing the abortion. This strategy would be easier to defend before God as well as the community.

The service-providing organizations that we talked to were troubled by the problems of disseminating knowledge and creating awareness of the guideline: *‘Because of its sensitive nature, advocacy is not being done. We cannot gather people and tell them this is the new guideline; it is difficult to share the guideline with the media’*. (INGO) Accordingly, dissemination mainly takes place through training of health workers who receive a copy of the guideline and take it home to share with their colleagues.

According to one of our informants in the INGO sector a major problem is that the law is included in the criminal code:

The law becomes a hindrance in itself. We have asked for safe abortion rooms in the region where we are working, but they refused because the law didn’t allow it since it has some prohibitions. If you open up a safe abortion room or publicly announce about it, people assume that you are encouraging it. (INGO)

The ambiguity of the law and the lack of knowledge and public debate about it was also said to hinder documentation of abortion services:

The hidden nature and restrictions make it difficult to have national data. I have seen a receipt of an acquaintance who accessed abortion services in a private hospital and it reads medical check-up. I do not know about government hospitals, but in the private ones it is hidden – they don’t write explicitly. (UN)

The commitment of the government to take responsibility for the roll-out of services into rural areas was also questioned. Some informants were concerned that in this highly conservative religious and cultural context *‘the government is reluctant to promote safe abortion and decentralize the agenda’*. It was observed that the abortion issue was commonly avoided in the regional health system review and planning meetings and hence *‘they will not be able to allocate budget for abortion care service’*. (INGO) Availability of the service out of urban centres was seen as a continued problem: ‘*the services should be available at the health centre level but only a few of them are providing the services. The law is here, but it is up to the NGOs to expand the services’*. (INGO) From our informants in the NGO sector we learned that international NGOs and UN agencies supported implementation of the law and the roll-out of services in a variety of ways. The assistance ranged from providing material support for abortion and post-abortion care services, training public sector health workers in clinical abortion and post-abortion care skills, operating private clinics to supplement public services (in the case of FGEA and Marie Stopes) or supporting local governmental and non-governmental organizations working on safe abortion.

The lack of information reaching potential service users has been acknowledged by the Ministry of Health, and the revised guidelines of 2014 took a new step in strengthening awareness of the law and access to services into the rural areas through the health extension programme. According to one of our UN informants, health extension workers now give information not only about access to safe abortion services but also *‘on legal issues like if she [the woman] goes to the health centres she needs to present reasons for abortion’*. (UN) This was regarded as an important step in strengthening access because presenting a reason that is outside the provisions of the law would exclude the woman from obtaining safe abortion services.

## Discussion

In sharp contrast to the contentious climate and the public debates taking place before the revision of the abortion law, there has been limited public attention to or visibility of the abortion law after it was enacted in 2004. It seems that questions about the law and access to safe abortion services may have been moved from the public sphere of debate to confined areas within organizations or groups of organizations sharing the same attitude. Through our analysis we identified ‘silence’ as a pro-active strategy used by several actors to avoid conflict and attention, but also as a protective reaction to the environment. As we have shown, the proponents of safe abortion used silence as a strategy to protect the law, while the religious organizations avoided conflict with the official stand of the government by confining their talk against the law to the religious arenas and community settings. Finally, the service-providing organizations used ‘the new Bible’ (the guidelines) to provide safe services but avoided advertising these services or communicating the number of abortion-seeking women so as not to attract unwanted attention in an environment where anti-abortion sentiments were widespread.

Drawing upon Walt and Gilson’s framework for policy analysis [15] and Shore and Wright’s perspectives [14] on the social life of polices, we will, in the following, discuss the international and national *context* and political climate before and after the revision of the abortion law; the role of the *actors* involved; and the *process* of implementation with particular emphasis on silence as a strategy. Secondly we will discuss the implications of silence as a strategy for access to safe abortion services, and will argue that despite its shortcomings, the alliance between the non-government and government actors to promote safe abortion without politicizing the agenda has been vital for the achievements made.

### The international and local context of the law and its implementation

When we explore the context within which it took place, the revision of the law clearly did not happen in an international vacuum. At the time Ethiopia relaxed its abortion law, religious conservativism and the anti-abortion pro-life movement was growing in influence globally, and represented a counter discourse to the reproductive health and rights discourse promoted in the International Conference on Population and Development [17] and its Programme of Action [18]. In 2001 President George W. Bush re-instated the Mexico City policy, known as the Global GAG rule cutting funds to organizations working to improve access to safe abortion [19]. This, according to Skuster [20], represented a set-back of the early initiatives to amend the abortion law in Ethiopia where major actors in reproductive health were funded by USAID. When the Millennium Development Goals (MDGs) were adopted following the United Nations Millennium Declaration in 2000 [21], universal access to reproductive health services, including prevention of unsafe abortion was not included [22]. MDG 5 was to reduce maternal mortality by three-quarters between 1990 and 2015 and the indicators included maternal mortality ratio and proportion of births attended by skilled health personnel [23]. Universal access to reproductive health was added in 2005 [24], but safe abortion as a tool to address maternal mortality was not even mentioned despite the fact that unsafe abortion was responsible for a substantial proportion of maternal deaths globally.

These contradictory discourses and concerns played into the law revision process and contributed to the compromise characterizing the law as it was enacted. The attainment of MDG 5 through reduced maternal mortality was indeed the argument used by the Ethiopian government to change the abortion law in a situation where abortion was estimated to account for 32% of the maternal deaths in the country [7]. At the same time, the operational guidelines, developed by the Ministry of Health after the revised law was passed in parliament in 2004, actively referred to women’s rights to health and choice, which are central to the ICPD Programme of Action [18]. Thus it seems that the government talked with several voices; one communicated that abortion is illegal, another that the prohibitions must be relaxed to save lives, and the third that women have choices and a right to decide over their own body. This ambiguity was reflected in the law itself. Keeping the abortion law in the penal code signaled that abortion is illegal, but the clause in the law that the woman’s word of rape or incest is enough to quality for safe abortion services opened up for choice and for positioning abortion as a reproductive rights issue. As Horn pinpointed in an article in Global Post [12], it may seem that the *actors* wanted to legalize abortion while making it appear illegal on paper in order to appease anti-abortion groups. This way the government appears to have succeeded in appeasing both camps [2]. The public health framing of the argument to change the law seems to have enabled policy makers including the Ministry of Health, health care providers and others involved to reconcile tensions between personal and religious values and the professional imperative to save lives.

When the law was passed, it attracted international attention as a liberal law in its context, and according to Wada [2] the change could not have happened without a strong political will. Given the position of Ethiopian society on sensitive issues like sexuality, sex work and homosexuality [4], the revision of the abortion law came as a surprise and points to the importance of central actors in the government. The late Prime Minister, Meles Zenawi, and the then Minister of Health, Tedros Adhanom Ghebreyesus, had the reduction of maternal mortality high on their political agenda (see e.g. [25]) and have been seen as instrumental in getting a more permissive law in place.

A decade after the law became operational, the anti-abortion movement seems to have limited foothold in Ethiopia compared to other countries in the region where ‘pro-life’ actors, funded through international networks, are more visible in anti-abortion advocacy (see e.g. the cases of Tanzania and Zambia this thematic series). Although there is a ‘pro-life Ethiopia network’ based in the US [26], the local response seems limited to scattered initiatives and campaigns (see e.g. [27]). The recent re-instatement of the global GAG rule by President Donald Trump in 2017 does not seem to have spurred a ‘pro-life’ surge within Ethiopia. This should be understood in the context of the action oriented authoritarian government in Ethiopia in office until 2018, known to ‘getting things done’ through a top-down approach and actively silencing dissenting voices or platforms. A clear expression of this is the Civil Society Law passed in 2009 which restrained civil society actors in doing advocacy for or against any agenda in reproductive health through cutting their access to funds from abroad to 10% [28]. As we have tried to illustrate in this paper, even the religious communities have not voiced a clear and coherent stand against the law, and we argue that the reason for their silence is the relative authority of the state vis a vis the church. In the authoritarian political context of Ethiopia, opposing the abortion law may not have been a productive strategy to maintain peaceful relations with the government. Though formally independent of the state, the Ethiopian Orthodox Tewahido Church has a history of interdependence with the Ethiopian government which still shapes their relationship today. This has also been the case with other church communities. Religious leaders have generally been appointed only if acceptable to the government, and there are examples of religious leaders being removed from their position or even imprisoned if perceived as a threat to the agenda of the government. In August 2015, eighteen Muslims including leaders were arrested and sentenced after months of peaceful protests, petitions, and appeals by the Muslim community against undue government interference in religious affairs [29].

Hence, a weak civil society curtailed by the Civil Society Law limiting international funding combined with a general absence of democratic experience, makes it difficult for religious and other non government actors to advocate against the course of action set by the authoritarian Ethiopian government.

But the absence of strong anti-abortion groups in Ethiopia does not reflect a positive attitude to safe abortion among the population at large. A comparative study in 2017 demonstrated that public opinion was not in line with the revised abortion law. It showed that members of EOTC are highly conservative on social issues compared to their co-believers in other parts of the world, and are much more inclined to state that “having an abortion is morally wrong” than Orthodox Christians in Central and Eastern Europe for example (83% vs. a median of 46%) [4]. Although this figure may vary with religious affiliation, it does indicate that public opinion on abortion in Ethiopia is still highly negative more than a decade after the revised abortion law was passed. A study of health workers in Tigray by contrast, reported that 55% of the participants supported women’s right to choose an abortion and that anti-abortion attitudes accounted for only 7% of the trained health practitioners [30]. This is most likely not representative for Ethiopia as a whole, but it gives an indication of health workers’ position as more open to abortion than the public at large. The same mechanisms that hinder active opposition to the existing abortion law, may also hinder the development of progressive social movements that enhance reproductive rights and challenge public opinion.

### Actors’ tacit alliance to prevent politicization

Here we return to the discussion of the central actors directly engaged in the policy implementation process, as framed by Walt and Gilson [14, 15]. These actors included the International NGOs involved in policy development, training and financial and infrastructural support (like IPAS), the service-providing organisations (like Marie Stopes International and Family Guidance Association of Ethiopia), the UN organisations providing assistance to the Ministry of Health (like WHO and UNFPA), and the Ministry of Health as policy maker and implementer. Although there were probably divisions of opinion within the Ministry of Health, the official stand was clearly to improve access to safe abortion services for eligible women. All these actors seemed to speak the same language and emphasised the commitment to working within the law and to doing it quietly so as not to provoke the public and cause politicization of the agenda, which in turn could cause a backlash. According to Scruton ([31]: 534), politicization refers to a process where a social phenomenon or an activity is transformed from having no ‘political connotations to one that is consciously bent towards political ends’, and which becomes the basis of mobilization and is turned into an issue of great political significance and conflict. Sexual and reproductive health, defined as deeply moral issues in society, are particularly susceptible to politicization, a pertinent example being the politicization of homosexuality and the re-enforcement of the colonial law prohibiting homosexual practices in Uganda in 2014 [32]. Other countries have followed, the most recent example being Tanzania [33]. In Ethiopia, there seems to be an implied or tacit agreement among these central actors to keep a low profile publicly and work effectively in silence. Silence thus emerged as a tactical strategy to increase access to safe abortion services, and to avoid the confrontational politics of abortion, which different actors in the field alluded to as unnecessary and counter-productive.

### Silence as a strategy and its implications for access

Silence can be pro-active and silence can be reactive, or to use Kenny’s [34] terms, silence can be either active or passive. Although our material exemplifies both, we argue that silence as a strategy has played a dominant role in the process of implementation in this case, and as a means of resisting or avoiding negative public opinion. Despite its downsides in terms of knowledge dissemination and advocacy, “the power of silence” [34] as an agentic strategy to extend access to safe abortion services in Ethiopia has been clearly demonstrated. Kenny notes that, “The more one speaks, the more one is inviting attention and perhaps an unfavourable or even dangerous response” ([34]: 17). Our informants involved in extending safe abortion services argued along the same lines, actively choosing to be silent in a bid to avoid negative public opinion against abortion.

But silence is not always an appropriate strategy and as articulated by Kenny, “.. silence may also be deadly……Political silences kill” ([34]: 9). The silent approach to abortion care in Ethiopia has left many women, health- and legal professionals in the dark about the abortion law and policy, and this has affected access to safe abortion services negatively. By and large, the level of public awareness about the law seems low and this hinders women’s access to safe abortion. A qualitative research study showed that awareness about the new liberalized abortion law was almost inexistent among the girls and women who participated in the study. This implied that their access to safe abortion care was also very limited – they did not know where to go for the service [35]. Further, in a quantitative study on awareness about the abortion law, 54.5% percent of the respondents did not know about the revised law [36].

As silence has led to lack of information to the general public, it has at the same time given health workers greater room for discretion. As we have tried to show in this paper, health workers play an important role as gate keepers regulating access to safe abortion services and can use their discretion both to facilitate and to prevent women from accessing services. On the one hand, they may interpret the law and implement the guidelines flexibly to support a woman’s decision to terminate pregnancy, claiming it is a result of rape or that the woman seeking abortion is a minor. Some of these providers seemed to understand that the law made abortion accessible ‘almost on demand’. Conversely, the health worker may himself or herself be ignorant about the law or be highly religious and conservative on family issues including abortion. In both cases the abortion-seeking woman may be denied services that she, according to the law, is entitled to. In this way, health workers are located at the core of implementation and represent the key to extending access to eligible women, particularly in the context of silence. In both instances, the silent approach stifles struggles for reproductive health rights. Even if the law is permissive, the practice may continue to be highly restrictive when girls and woman are not aware of their rights to services.

Through a scrutinization of the actors engaged in the policy implementation processes, and the context within which the new abortion law has been implemented, we have seen that silence as a strategy has prevented a public debate that could help reduce the stigma surrounding abortion. In other words, by employing silence as a strategy, the actors may have managed to expand access to safe abortion and saved women’s lives, but may at the same time have curtailed public debate about gendered, social, moral, economic and legal issues that expose women to unwanted pregnancy and unsafe abortion. The inherent complexity of the policy dynamics at work reveal the social life of policy as detailed by Shore and Wright [13, 14].

Overall, different studies conducted on the impact of the law and our qualitative data show that the new law has substantially increased access to safe abortion care by expanding the grounds on which it can be granted. Nonetheless unsafe abortion and inequity of access still remain high. Particularly, barriers are encountered by rural women who represent the highest proportion of women of reproductive age, and who are more likely to have unsafe abortions and accompanying serious complications than urban women. This suggests that there is critical need for continued expansion of affordable abortion services in public facilities to reach underserved groups such as the young, rural communities and/or women in peripheral regions of the country. The involvement of health extension workers as bridges to safe abortion services as described in the revised guidelines of 2014 [37] is another indication of the political will to improve access to services. How this will work in practice, i.e. how health extension workers will manage to fit legal advice-giving into their already overburdened everyday work is not yet known.

Changing social norms and public opinion around abortion requires a different approach that actively engages with values at the community level as well as on the health worker level.

Abiy Ahmed entered office as Prime Minister in Ethiopia in 2018 aiming for a more open democratic society. The new Civil Society Law of 2018, lifting restrictions on funding and advocacy, facilitates the development of a stronger civil society and grassroots movements. This lays the ground for two scenarios. With the appointment of 50% women in the cabinet and other key positions in government, a woman centred, rights based approach to abortion services may gradually come to complement the public health approach that has been vital to extend safe abortion services for the last decade. This would be in line with the human rights basis of the Ministry of Health Guidelines to safe abortion care of 2006 [7] and 2014 [37]. The other side of the coin is that the Civil Society Law, in the context of the anti abortion movement in the US coupled with the Global GAG rule, may lay new grounds for anti abortion actors. In the longer term perspective this can threaten the revised abortion law of 2004, and the achievements made in improving access to safe abortion services.

### Study limitations

The interviews that the study findings are based on were conducted at three points in time between November 2016 and April 2018 with the purpose of investigating how the revised abortion law had fared after it was made operational through the MOH guidelines published in 2006 and 2014. This implied that we did not follow the implementation of the law from the beginning and had to rely on retrospective interviews with actors and their memory and interpretation of the process. We were particularly interested in how the main actors, both governmental and non-governmental, developed strategies for implementation, and how these strategies affected access to services for eligible women. We did, however, not collect qualitative or quantitative information from users on access, so our discussion is based on the impressions, experiences and observations of the actors involved in the process of revising the law and implementing safe abortion services. This is obviously a design weakness since these actors may have an interest in success and may have exaggerated the positive outcomes. However, there are quantitative studies that describe the same trend and support these actors' position as represented in our findings. An archival study to supplement the interview data would have been useful, but was not possible to do within the time frame of the study. We may have missed out on important information and events related to the process of implementing the law such as anti-abortion activities which we may have underestimated. However, the number and centrality of the organizations included, some of which have a very long history in the field, and the repeat interviews with core actors and triangulation with previous quantitative and qualitative studies strengthen the study findings and conclusions.

## Conclusion

The implementation of the Ethiopian abortion law can only be understood with a recognition of the ambiguities inherent in the law and the opportunities for interpretation that this involves. A liberal interpretation of the law may be threatened by a public debate around the provisions and clauses of the law. Such debate could also threaten the very existence of the law and this is what ‘tacitly aligned’ actors fear the most. Therefore, if the central actors talk about abortion in public at all, it is framed in the discourse of public health or saving the life of a dying woman. In this way, the actors seem to be accommodative of existing public opinion. They seem to have decided to keep quiet while seizing the opportunity to provide services to the full extent of the law. At the same time silence seems to have helped actors resolve the tension between local social and religious values, the medical necessity to save lives, and local and international actors’ interest in complying with international frameworks on sexual and reproductive health and rights (SRHR) issues. Thus, the silent approach is a good entry point, but cannot be a sufficient long-term response to the abortion issue since it does not promote awareness of and access to safe abortion services, and does not challenge existing norms and values. We conclude with Horn [11] that ‘dealing with abortion in Ethiopia means dealing with contradiction. But in a land of many paradoxes, such contradictions can persist far longer than they might in a more open society. For now, at least, the uneasy compromise remains in place’.

## Data Availability

To protect the study participants’ anonymity (some representatives of small NGO’s) the interview data generated and analyzed during the current study is not publicly available. The data may be made available from the corresponding author on reasonable request.
